# Fructose Consumption by Adult Rats Exposed to Dexamethasone In Utero Changes the Phenotype of Intestinal Epithelial Cells and Exacerbates Intestinal Gluconeogenesis

**DOI:** 10.3390/nu12103062

**Published:** 2020-10-07

**Authors:** Gizela A. Pereira, Frhancielly S. Sodré, Gilson M. Murata, Andressa G. Amaral, Tanyara B. Payolla, Carolina V. Campos, Fabio T. Sato, Gabriel F. Anhê, Silvana Bordin

**Affiliations:** 1 Department of Physiology and Biophysics, Institute of Biomedical Sciences, University of Sao Paulo, Sao Paulo, 05508-000 SP, Brazil; gizela.usp@gmail.com (G.A.P.); frhanshirley@gmail.com (F.S.S.); gilmasa@gmail.com (G.M.M.); andressa_amaral@usp.br (A.G.A.); tany_line@yahoo.com.br (T.B.P.); fabiotakeosato@gmail.com (F.T.S.); 2 Department of Pharmacology, Faculty of Medical Sciences, State University of Campinas, Campinas, 13083-887 SP, Brazil; ca.v.campos20@gmail.com (C.V.C.); anhegf@unicamp.br (G.F.A.)

**Keywords:** intrauterine growth restriction (IUGR), fructose, dexamethasone, intestinal gluconeogenesis

## Abstract

Fructose consumption by rodents modulates both hepatic and intestinal lipid metabolism and gluconeogenesis. We have previously demonstrated that in utero exposure to dexamethasone (DEX) interacts with fructose consumption during adult life to exacerbate hepatic steatosis in rats. The aim of this study was to clarify if adult rats born to DEX-treated mothers would display differences in intestinal gluconeogenesis after excessive fructose intake. To address this issue, female Wistar rats were treated with DEX during pregnancy and control (CTL) mothers were kept untreated. Adult offspring born to CTL and DEX-treated mothers were assigned to receive either tap water (Control-Standard Chow (CTL-SC) and Dexamethasone-Standard Chow (DEX-SC)) or 10% fructose in the drinking water (CTL-fructose and DEX-fructose). Fructose consumption lasted for 80 days. All rats were subjected to a 40 h fasting before sample collection. We found that DEX-fructose rats have increased glucose and reduced lactate in the portal blood. Jejunum samples of DEX-fructose rats have enhanced phosphoenolpyruvate carboxykinase (PEPCK) expression and activity, higher facilitated glucose transporter member 2 (GLUT2) and facilitated glucose transporter member 5 (GLUT5) content, and increased villous height, crypt depth, and proliferating cell nuclear antigen (PCNA) staining. The current data reveal that rats born to DEX-treated mothers that consume fructose during adult life have increased intestinal gluconeogenesis while recapitulating metabolic and morphological features of the neonatal jejunum phenotype.

## 1. Introduction

The consumption of fructose-sweetened beverages has significantly increased during the last decades and a great number of observational studies have associated this nutritional habit with increased cardiometabolic risk [1]. In accordance with this hypothesis, experimental studies have described that rats consuming high amounts of fructose or sucrose develop glucose intolerance and increased hepatic gluconeogenesis [2,3,4].

The mechanisms underlying the metabolic effects of excessive fructose intake rely on its hepatic as well as its intestinal metabolism [5]. The small intestine absorbs fructose through a facilitated glucose transporter member 5 (GLUT5)-dependent mechanism and partially metabolizes it into lactate, glucose, and fatty acids that are sequentially secreted to the portal circulation [6,7]. However, intestinal fructose metabolism capacity is limited and when high amounts of fructose are consumed, a considerable fraction reaches the liver [7]. Hepatic metabolism of fructose, in turn, produces glyceraldehyde-3-phosphate that is driven to either gluconeogenesis or de novo lipogenesis (DNLG) [8].

Besides its metabolism to intermediates that feed gluconeogenesis and DNLG, fructose was described to modulate the expression of key metabolic genes [5]. Fructose consumption increased the expression of GLUT5 and gluconeogenic enzymes glucose-6-phosphatae (G6Pase) and fructose-1,6-bisphosphatase (FBP1) in the small intestine [9,10]. Excessive fructose intake was also reported to increase the hepatic expression of the gluconeogenesis enzymes G6Pase, FBP1, and phosphoenolpyruvate carboxykinase (PEPCK) [11,12,13,14], and the DNLG enzymes acetyl-CoA carboxylase (ACC), fatty acid synthase (FAS), and stearoyl-CoA desaturase-1 (SCD1) [15].

We have recently demonstrated that the modulation of key metabolic genes induced by fructose in the liver can interact with other factors, such as birth weight. Fructose supplementation of rats born small due to maternal treatment with dexamethasone (DEX) induced an expected increase in the expression of PEPCK, FAS, and ACC, but failed to increase the expression of genes involved in very low density lipoproteins (VLDL) assembly and secretion, leading to an exacerbation in hepatic steatosis [16].

In addition to low birth weight, in utero exposure to DEX is recognized to program the energy metabolism during adult life. Offspring born to DEX-treated mothers develop glucose intolerance and increased hepatic PEPCK expression as soon as the 21st day of life [17]. Additionally, pancreatic postnatal development of rats born to DEX-treated mothers is hallmarked by lower pancreatic b-cell mass and higher pancreatic a-cell mass and glucagon levels [18]. Treatment of pregnant mice with DEX was also described to epigenetically impair brown adipose tissue (BAT) thermogenesis and energy expenditure in the offspring, leading to increased adiposity and insulin resistance [19].

Aside from the liver, several studies have reported the expression of G6Pase and PEPCK in the small intestine of humans and rats, deeming this organ relevant in endogenous glucose production (EGP) [20,21,22]. Significant contribution of the small intestine to EGP is particularly relevant after a fasting period of at least 40 h, the period of time necessary for an increase in both G6Pase and PEPCK in the jejunum [23,24,25].

The present study has been undertaken to evaluate if prenatal exposure to DEX and excessive consumption of fructose during adult life could interact to modulate small intestine gluconeogenesis. To achieve this aim, we have evaluated key enzymatic and biochemical end-points indicative of intestinal gluconeogenesis as well as morphological aspects of the jejunum in 40 h fasted rats born to DEX-treated mothers and/or exposed to liquid fructose during adulthood.

## 2. Materials and Methods

### 2.1. Experimental Design and Diet

Eight-week-old nulliparous Wistar rats were acquired from the Animal Breeding Center at the Institute of Biomedical Sciences, University of Sao Paulo (Protocol # 5367250619). The animals were housed and mated with male rats as previously described [16].

After mating, pregnant rats were randomly assigned to receive 0.1 mg/kg/day dexamethasone (DEX) diluted in the drinking water from the 14th to the 19th day of pregnancy or remain untreated (CTL). On the 80th day of life, male offspring of DEX-treated and CTL dams were divided into two additional groups that were either kept with tap water or 10% fructose (w/v) solution ad libitum for the next 80 days. All offspring received standard chow ad libitum from the weaning to the 160th day of life.

The different groups were thereafter designed as follows: offspring born to CTL mothers that received only standard chow (SC) and tap water during adult life (CTL-SC); offspring born to DEX-treated mothers that received only SC and tap water during adult life (DEX-SC); offspring born to CTL mothers that received SC plus 10% fructose during adult life (CTL-fructose); and offspring born to DEX-treated mothers that received SC plus 10% fructose during adult life (DEX-fructose). On the 160th day of life, the animals were subjected to a 40 h fasting before euthanasia. During fasting, standard chow was removed and 10% fructose was replaced by tap water.

### 2.2. Pyruvate Tolerance Test (PTT)

Rats were fasted for 40 h and a 20% sodium pyruvate solution was injected intraperitoneal (i.p.) at a dosage of 2 g/kg of body mass. Glucose concentration was determined in blood extracted from the tail before (0 min) and 15, 30, 60, and 90 min after pyruvate injection (tail blood samples were chosen as representative of systemic blood). The area under the curve (AUC) of tail blood glucose levels vs. time was calculated using each individual baseline (basal glycemia) to estimate whole-body gluconeogenesis. We also collected portal blood samples at the end of the PTT (90 min after pyruvate challenge) to estimate the ability of the small intestine to convert pyruvate into glucose.

### 2.3. Tissue Sampling and Preparation

The rats were anesthetized with isoflurane. Proper level of anesthesia was assured by loss of pedal reflex. The abdominal cavity then was opened and portal blood was punctured. Euthanasia was performed by rupture of the diaphragm (with surgical scissors) followed by immediate cardiac puncture of the systemic blood. Systemic and portal blood samples were collected in EDTA-coated tubes and centrifuged at 2000 rpm for 20 min at 4 °C. Plasma samples were removed and used for biochemical analysis.

Intestinal segments were harvested from the proximal jejunum (5 cm beyond the ligament of Treitz), opened at the mesenteric border, pinned flat on a cork mat, and gently washed in ice-cold 0.1 M phosphate-buffered saline solution (PBS). The time elapsed between euthanasia and jejunum samples harvesting was approximately 2 min. The segments were transversally cut into two samples. In one sample, the mucosa was scraped and frozen under −80 °C for subsequent molecular and biochemical analyses (described below).

The second jejunum sample was immediately fixed with 10% buffered formaldehyde for 24 h, dehydrated in alcohol, diaphanized in xylol, and embedded in paraffin. Nonserial longitudinal sections (5–7 μm thick) were subjected to hematoxylin–eosin (HE) staining for morphometric analysis. In some rats, the entire small intestine was dissected and its length (cm) was measured and expressed as the relative length to tibia length [26].

### 2.4. Analysis of Blood Parameters

Plasma glucose, triglycerides, lactate, and cholesterol determinations were performed using commercially available kits (Labtest Diagnóstica SA, Lagoa Santa, MG, Brazil).

### 2.5. Enzymatic Activity

The activities of phosphoenolpyruvate carboxykinase (PEPCK; EC 4.1.1.32), glucose-6-phosphatase (G6Pase; EC 3.1.3.9), and hexokinase (HK; EC 2.7.1.1) were measured using spectrophotometric assays at 340 nm, following standard methods described elsewhere [27,28,29]. Protein concentration of each sample was determined by the Bradford method, and enzymatic activities were normalized by protein content.

### 2.6. Molecular Analyses

Scraped mucosal cells were processed for both qPCR and Western blotting (WB), as previously described [16]. The nitrocellulose membranes for WB were stained with Ponceau S before incubation with the primary antibodies. The stained membranes were allowed to dry at room temperature, scanned, and subjected to optical density (OD) quantification. All the lanes (from the top to the bottom) of labeled proteins were scanned to better represent the total amount of protein actually loaded in the gel. Subsequently, these values were applied to normalize the OD data of the target proteins detected in the respective membranes. This method was validated as an appropriate loading control [30]. The primary antibodies used were as follows: anti-GLUT2 (cat. # sc-9117) from Santa Cruz Biotechnology (Santa Cruz, CA, USA) and anti-GLUT5 (cat. # IM-0292) from Rhea Biotech (Campinas, SP, Brazil).

Total RNA was extracted using QIAzol reagent and used for reverse transcription with random primers for the analysis of mRNA expression. The primer sequences and accession numbers were as follows: *G6pc* (NM_013098) 5′-ACCTTCTTCCTGTTTGGTTTCGC-3′ and 5′-CGGTACATGCTGGAGTTGAGGG-3′; *Pck1* (NM_198780) 5′-TGGTCTGGACTTCTCTGCCAAG-3′ and 5′-AATGATGACCGTCTTGCTTTCG-3′; *Ggt1* (NM_053840) 5′-ACCCGACTTCATCGCTGTG-3′ and 5′-GCATGTTCTCCAGAGTCCCAC-3′; *Rpl37a* (X14069) 5′-CAAGAAGGTCGGGATCGTCG-3′; and 5′-ACCAGGCAAGTCTCAGGAGGTG-3′. Values of mRNA expression were normalized using the internal control gene *Rpl37a*. Fold changes were calculated by the 2^−ΔDDCT^ method.

### 2.7. Morphometric Analysis of the Jejunum Wall

The morphometric analyses were performed blindly using AxioVision Release 4.8-SP2 software (Carl Zeiss Microscopy, Jena, Germany) and consisted of the evaluation of the villus height and crypt depth. The height of each villus was measured from the top of the villus to the crypt transition, and the crypt depth was defined as the invagination between two villi. These analyses were performed in five fields at 100× magnification from different jejunum regions, including 2–3 villus/crypt per field, totaling 10–15 villus/crypt per animal [31].

### 2.8. Immunohistochemical Evaluation of Cell Proliferation

Antigen retrieval was performed in citric acid (10 mM, pH 6.0) at 95 °C for 40 min, followed by cooling for 30 min. After antigen retrieval, sections were incubated with 3% hydrogen peroxide diluted in methanol for 30 min to quench endogenous peroxidase, then rinsed with deionized water followed by PBS, pH 7.4. Sections then were incubated with 6% defatted milk for 30 min, at 37 °C, to block nonspecific staining. The anti-PCNA monoclonal antibody, from Dako-Agilent (Santa Clara, CA, USA; Cat. No. M0879), was diluted 1:1000 in PBS plus 1% bovine serum albumin (BSA) and incubated on sections overnight at 4 °C. All sections were washed three times in PBS for 5 min each time, then incubated with secondary antibody conjugated with horseradish peroxidase labeled polymer (EnVision + Dual link System-HRP) for 30 min at room temperature. 3,3′-diaminobenzidine (DAB) was used to visualize the antigen/antibody complex and the specimens were then lightly counterstained with hematoxylin. Negative control samples were performed by substituting the primary antibody with antibody diluent. We analyzed 10 crypts from each section under a 400× magnification [32]. Cell proliferation rate was expressed as percentage of PCNA-positive cells.

### 2.9. Statistical Analyses

Comparisons were performed using two-way ANOVA, followed by a Tukey’s multiple comparison test. The two factors considered for the two-way ANOVA were in utero exposure to DEX (either exposed or not) and treatment of 10% fructose during adulthood (either treated or not). When making comparisons between two groups, the unpaired Student’s *t*-test was used. Statistical analyses were conducted using GraphPad Prism software version 8.4.3 (GraphPad Software, Inc., San Diego, CA, USA). All results are presented as the means ± standard error of the mean (SEM). Results with *p* values lower than 0.05 were considered significant.

## 3. Results

### 3.1. Consumption of Fructose by Rats Born to DEX-Treated Mothers Modifies Body Composition but Does Not Modulate Small Intestine Length

**Figure 1:**
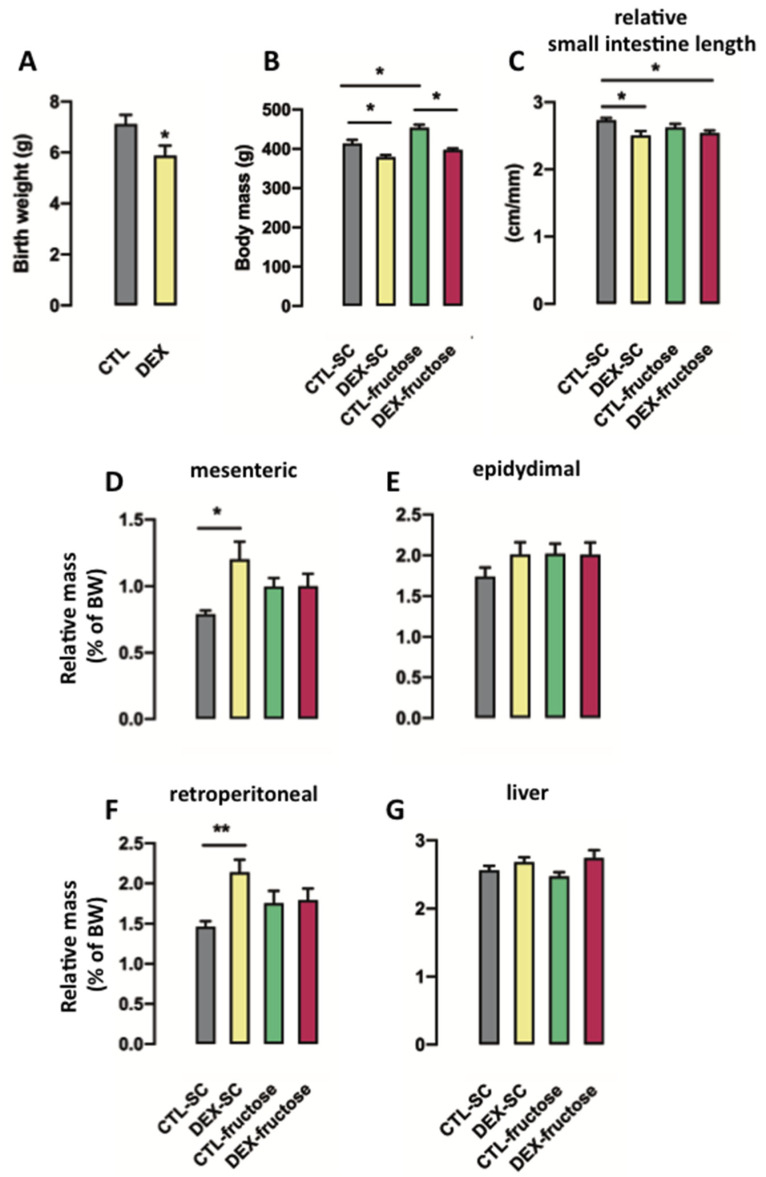
Morphometrical parameters of rats exposed to dexamethasone (DEX) in utero. Body weight was measured at birth (**A**) and at the end of treatment (**B**) in nonfasted rats. Fasted rats were euthanized at the end of the 80th day of fructose consumption, and the small intestine length relative to tibia length (**C**), and fat pads (mesenteric, (**D**); epidydimal, (**E**); retroperitoneal, (**F**)) and liver (**G**) masses relative to body weight were also measured. Results are presented as mean ± standard error of the mean (S.E.M.). * *p* < 0.05, ** *p* < 0.01 (*n* = 10–20). Offspring born to control (CTL) mothers that received only standard chow (SC) and tap water during adult life (CTL-SC); offspring born to DEX-treated mothers that received only SC and tap water during adult life (DEX-SC); offspring born to CTL mothers that received SC plus 10% fructose during adult life (CTL-fructose); and offspring born to DEX-treated mothers that received SC plus 10% fructose during adult life (DEX-fructose).

As reported by us in previous studies [16,33], rats born to DEX-treated mothers displayed reduced birth weight (18% lower than CTL; *p* = 0.033) (Figure 1A). The body weights of the 40 h fasted offspring with 160 days of age were influenced by both in utero exposure to DEX and treatment with 10% fructose (*p* < 0.0001 and *p* = 0.0002, respectively). The post hoc analysis revealed that DEX-SC were lighter (9%; *p* < 0.01) while CTL-fructose were heavier (10%; *p* < 0.001) when compared to age-matched CTL-SC. In addition, DEX-fructose rats were lighter than the SC-fructose group (13%; *p* < 0.0001) (Figure 1B).

Small intestine length, relative to tibia length, was influenced by in utero exposure to DEX (*p* = 0.0035). The post hoc analysis revealed that both DEX-SC and DEX-fructose had shorter small intestine when compared to CTL-SC (respectively 8% and 7% shorter; *p* < 0.05) (Figure 1C).

Mesenteric adiposity was influenced by the factor in utero exposure to DEX in the 40 h fasted offspring (*p* = 0.0264). However, the post hoc analysis revealed a specific increase of mesenteric adiposity in 40 h fasted DEX-SC (42% higher than CTL-SC; *p* < 0.05) (Figure 1D). The treatments had no effect on epididymal adiposity (Figure 1E). Retroperitoneal adiposity of the 40 h fasted adult offspring presented changes that were similar to those seen for mesenteric adiposity. The post hoc analysis revealed a specific increase of retroperitoneal adiposity of the 40 h fasted DEX-SC (50% higher than CTL-SC; *p* < 0.01) (Figure 1F). The relative weight of the liver was not affected by the treatments (Figure 1G).

### 3.2. Biochemical Changes Detected in Rats Born to DEX-Treated Mothers That Consume Fructose during Adulthood Indicates Increased Intestinal Gluconeogenesis

**Figure 2:**
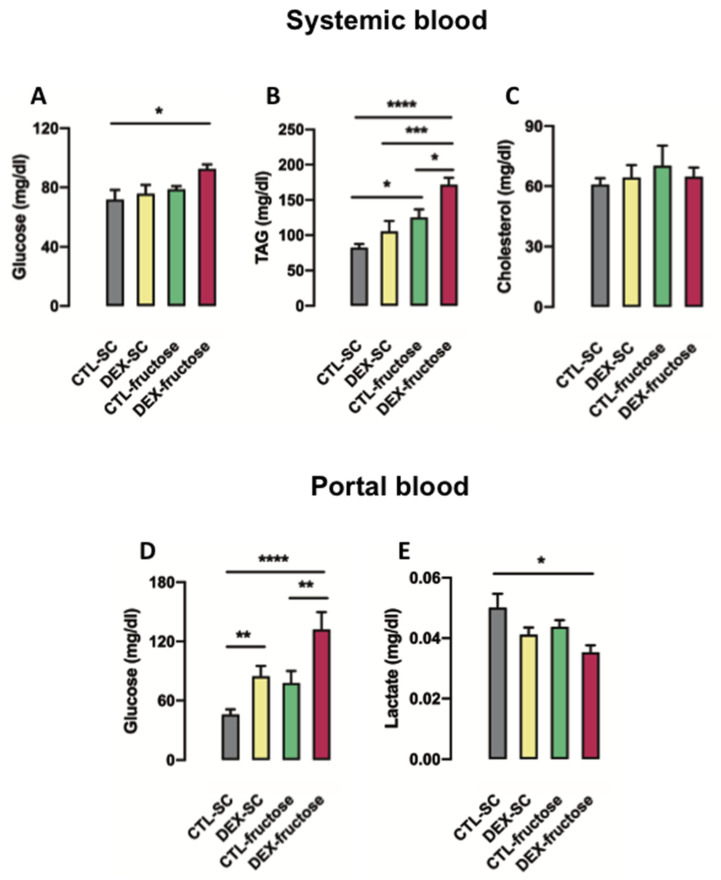
Effects of fructose on biochemical parameters of systemic and portal blood in rats exposed to dexamethasone (DEX) in utero. Systemic blood samples were collected to measure glucose (**A**), triacylglycerol (**B**), and total cholesterol (**C**). Portal hepatic vein samples were collected to measure glucose (**D**) and lactate (**E**). Results are presented as mean ± standard error of the mean (S.E.M.). * *p* < 0.05, ** *p* < 0.01, *** *p* < 0.001, **** *p* < 0.0001 (*n* = 8–12). Offspring born to control (CTL) mothers that received only standard chow (SC) and tap water during adult life (CTL-SC); offspring born to DEX-treated mothers that received only SC and tap water during adult life (DEX-SC); offspring born to CTL mothers that received SC plus 10% fructose during adult life (CTL-fructose); and offspring born to DEX-treated mothers that received SC plus 10% fructose during adult life (DEX-fructose).

Both in utero exposure to DEX and treatment with 10% fructose during adulthood affected systemic glucose levels after a 40 h fasting (*p* = 0.046 and *p* = 0.039, respectively). However, the post hoc analysis revealed that systemic glucose levels were increased exclusively in 40 h fasted DEX-fructose (28% higher than CTL-SC; *p* < 0.05) (Figure 2A). Similarly, in utero exposure to DEX and treatment with 10% fructose during adulthood influenced systemic triglyceride levels after a 40 h fast (*p* = 0.0025 and *p* < 0.0001, respectively). In regard to this, the post hoc analysis indicated that systemic triglyceride levels were increased in 40 h fasted DEX-fructose when compared to CTL-SC, DEX-SC, and CTL-fructose (respectively 108%, 62%, and 36%; *p* < 0.0001, *p* < 0.001, and *p* < 0.05). Systemic triglycerides were also increased in 40 h fasted CTL-fructose (52% higher than CTL-SC; *p* < 0.05) (Figure 2B). Total systemic cholesterol levels after 40 h fasting were not altered in any of the four groups studied (Figure 2C).

Both in utero exposure to DEX and treatment with 10% fructose during adulthood also modified portal glucose levels after a 40 h fasting (*p* < 0.0001 and *p* = 0.045, respectively). Our post hoc analysis revealed that portal glucose levels were increased in 40 h fasted DEX-fructose (144% higher than CTL-SC and 83% higher than CTL-fructose; *p* < 0.0001 and *p* < 0.01). The portal glucose levels were also increased in 40 h fasted DEX-SC (102% higher than CTL-SC; *p* < 0.05) (Figure 2D). Portal lactate levels detected after a 40 h fasting were only influenced by in utero exposure to DEX (*p* < 0.05). The post hoc analysis revealed a specific reduction of portal lactate levels in 40 h fasted DEX-fructose rats (26% lower than CTL-SC; *p* < 0.05) (Figure 2E).

### 3.3. Rats Born to DEX-Treated Mothers That Consume Fructose during Adulthood Display Increased Portal Glucose Levels after Challenge with Exogenous Pyruvate

**Figure 3:**
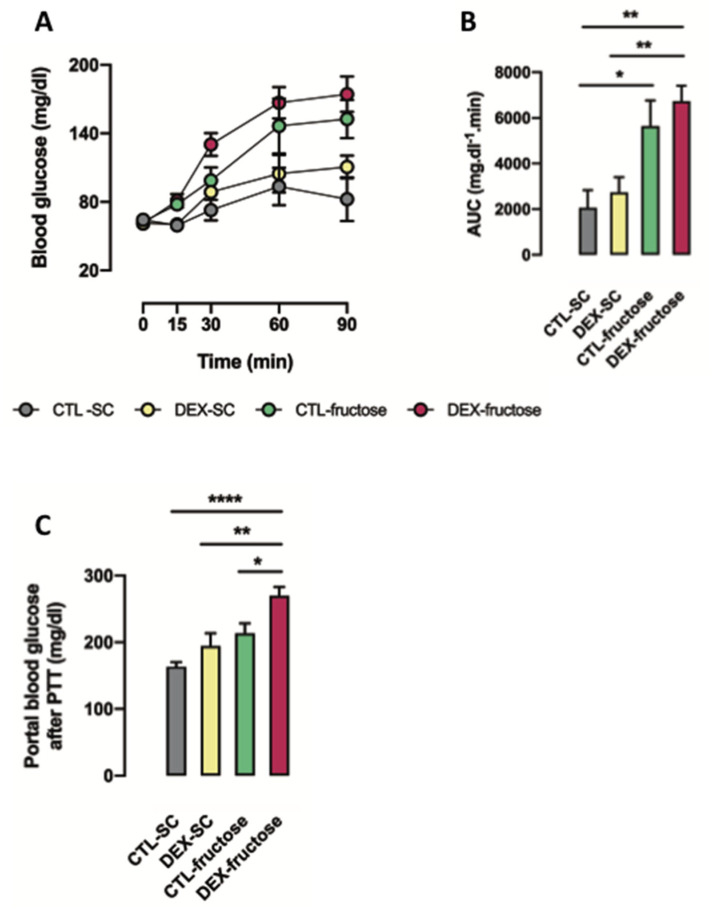
Whole-body and intestinal use of pyruvate as a gluconeogenesis substrate by rats exposed to dexamethasone (DEX) in utero and treated with fructose during adult life. The 40 h fasted rats received an i.p. injection containing sodium pyruvate. The blood from the tail was collected before and 15, 30, 60, and 90 min after intraperitoneal (i.p.) injection for glucose measurements (**A**) and the area under the curve (AUC) was calculated above each individual baseline (**B**). Glucose levels were also measured in portal blood at the end of the pyruvate tolerance test (PTT) (**C**). Results are presented as mean ± standard error of the mean (S.E.M.) * *p* < 0.05, ** *p* < 0.01, **** *p* < 0.0001 (*n* = 8). Offspring born to control (CTL) mothers that received only standard chow (SC) and tap water during adult life (CTL-SC); offspring born to DEX-treated mothers that received only SC and tap water during adult life (DEX-SC); offspring born to CTL mothers that received SC plus 10% fructose during adult life (CTL-fructose); and offspring born to DEX-treated mothers that received SC plus 10% fructose during adult life (DEX-fructose).

We next performed the pyruvate tolerance test with the attempt to clarify if the higher portal glucose levels seen in 40 h fasted DEX-fructose rats were due to increased gluconeogenesis. The glucose levels in tail blood samples were assessed at different time points after pyruvate injection (Figure 3A). Treatment with 10% fructose during adulthood influenced the area under the curve (AUC) values in 40 h fasted rats (*p* < 0.0001). Our post hoc analysis indicated that whole-body gluconeogenesis is increased in fructose-treated rats irrespective of maternal treatment with DEX. This can be concluded because the AUC values of both CTL-fructose and DEX-fructose rats were similar to each other and higher than those of CTL-SC (respectively 174% and 227% higher; *p* = 0.0227 and *p* = 0.0016) (Figure 3B).

Glucose levels in portal blood 90 min after challenge with pyruvate were influenced by both in utero exposure to DEX and treatment with 10% fructose during adulthood (*p* = 0.0043 and *p* = 0.0001, respectively). In contrast to the changes in whole-body gluconeogenesis, our post hoc analysis revealed increased portal glucose levels after challenge with pyruvate exclusively in DEX-fructose rats (65% higher than CTL-SC, 26% higher than CTL-fructose, and 39% higher than DEX-SC; *p* < 0.001, *p* = 0.0301, and *p* = 0.0055) (Figure 3C).

### 3.4. Rats Born to DEX-Treated Mothers That Consume Fructose during Adulthood Display Increased PEPCK Expression and Activity in the Jejunum

**Figure 4:**
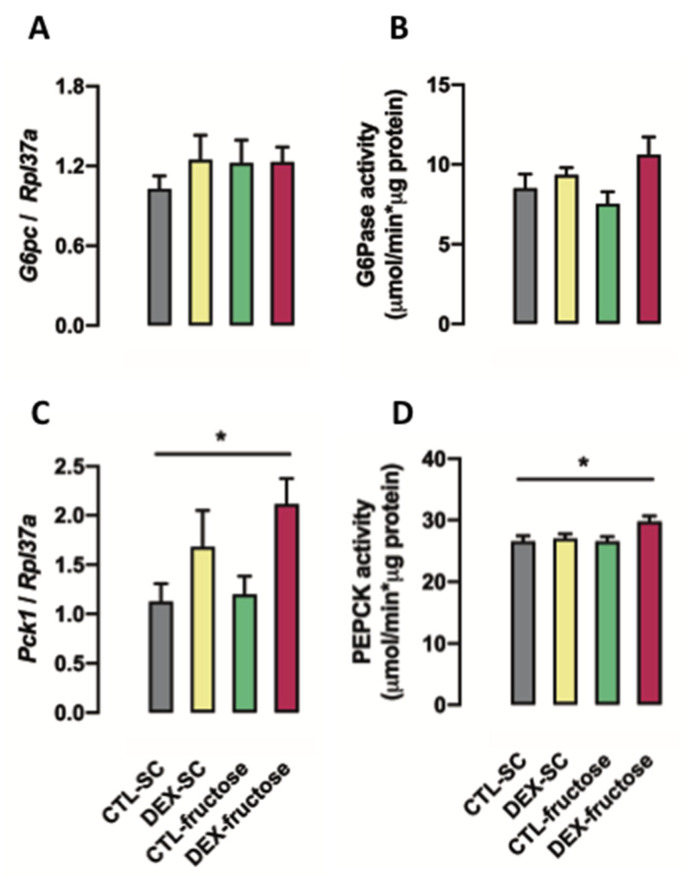
Expression and activity of enzymes involved in intestinal gluconeogenesis. Scraped epithelium of jejunum fragments was isolated and processed for qPCR detection of G6pc (**A**) and Pck1 (**C**) gene expression, as well as for maximum activities of the corresponding enzymes G6Pase (**B**) and PEPCK (**D**). Results are presented as mean ± standard error of the mean (S.E.M.). * *p* < 0.05 (*n* = 6–12). Offspring born to control (CTL) mothers that received only standard chow (SC) and tap water during adult life (CTL-SC); offspring born to dexamethasone (DEX)-treated mothers that received only SC and tap water during adult life (DEX-SC); offspring born to CTL mothers that received SC plus 10% fructose during adult life (CTL-fructose); and offspring born to DEX-treated mothers that received SC plus 10% fructose during adult life (DEX-fructose).

The expression of *G6pc* (the gene that encodes G6Pase) in the jejunum was not affected in any of the four groups after a 40 h fasting (Figure 4A). The activity of G6Pase in the jejunum of the 40 h fasted rats was influenced by in utero exposure to DEX (*p* = 0.035) but no specific differences were found in the post hoc analysis (Figure 4B).

The expression of *Pck1* (the gene that encodes PEPCK) in the jejunum of the 40 h fasted rats was modulated by in utero exposure to DEX (*p* = 0.005). In this case, our post hoc analysis indicated a marked increase of *Pck1* expression in the jejunum of the 40 h DEX-fructose (88% higher than CTL-SC; *p* < 0.05) (Figure 4C). As with changes in expression, PEPCK activity in the jejunum of the 40 h fasted rats was regulated by in utero exposure to DEX (*p* = 0.043). Our post hoc analysis indicated a specific increase of PEPCK activity in the jejunum of the 40 h fasted DEX-fructose group (14% higher than CTL-SC; *p* < 0.05) (Figure 4D). The expression of fructose 1,6-bisphosphatase (*Fbp1*) was also evaluated but no differences were found among the groups (data not shown).

### 3.5. Rats Born to DEX-Treated Mothers That Consume Fructose during Adulthood Display Reduced HK Activity and Increased GLUT5 and GLUT2 Expression in the Jejunum

**Figure 5:**
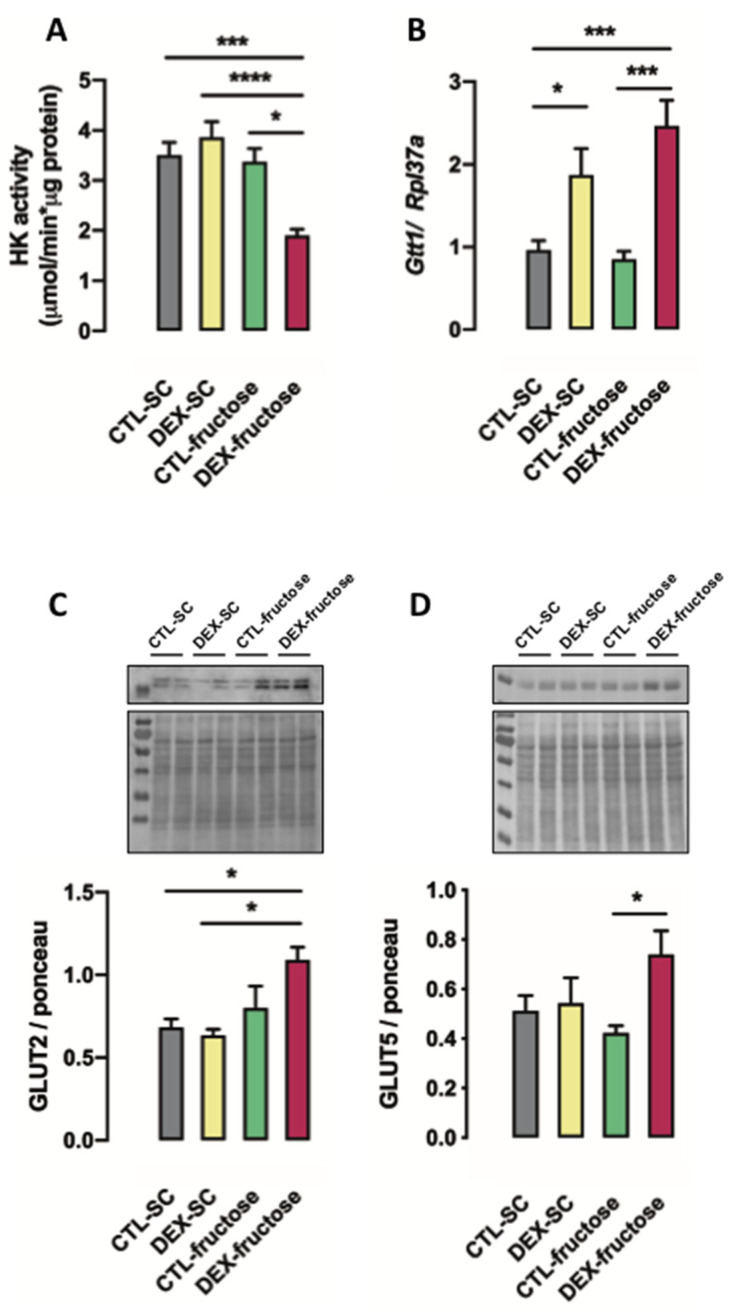
In utero dexamethasone (DEX) exposure alters glucose metabolism and phenotypic features of the jejunum epithelia. Scraped epithelium of jejunum fragments was isolated and processed for measurement of maximum hexokinase activity (**A**), expression of glutathione S-transferase 1 (Ggt1) by quantitative polymerase chain reaction (qPCR) (**B**), and Western blot of facilitated glucose transporter member 2 (GLUT2) (**C**) and facilitated glucose transporter member 5 (GLUT5) (**D**). Results are presented as mean ± standard error of the mean (S.E.M.). * *p* < 0.05, *** *p* < 0.001, **** *p* < 0.0001 (*n* = 6–12). Offspring born to control (CTL) mothers that received only standard chow (SC) and tap water during adult life (CTL-SC); offspring born to DEX-treated mothers that received only SC and tap water during adult life (DEX-SC); offspring born to CTL mothers that received SC plus 10% fructose during adult life (CTL-fructose); and offspring born to DEX-treated mothers that received SC plus 10% fructose during adult life (DEX-fructose).

Both in utero exposure to DEX and treatment with 10% fructose during adulthood influenced hexokinase (HK) activity in the jejunum of the 40 h fasted rats (*p* = 0.0362 and *p* = 0.0004, respectively). The post hoc analysis revealed that reductions in HK activity in the jejunum of the 40 h fasted rats were specific for the DEX-fructose group (46% lower than CTL-SC, 51% lower than DEX-SC, and 44% lower than CTL-fructose; *p* < 0.001, *p* < 0.0001, and *p* < 0.01) (Figure 5A).

The expression of glutathione S-transferase 1 (*Gtt1*) in the jejunum of the 40 h fasted rats was influenced by in utero exposure to DEX (*p* < 0.0001). The post hoc analysis revealed that 40 h fasted DEX-SC rats had increased expression of *Gtt1* in the jejunum (90% higher than CTL-SC; *p* < 0.05). Increased expression of *Gtt1* in the jejunum was also found in 40 h fasted DEX-fructose (170% higher than CTL-fructose and 156% higher than CTL-SC; *p* < 0.001) (Figure 5B).

The content of facilitated glucose transporter member 2 (GLUT2) in the jejunum of the 40 h fasted rats was altered by treatment with 10% fructose during adulthood (*p* = 0.0017). The post hoc analysis revealed that the increase of GLUT2 content in the jejunum of the 40 h fasted rats was specific for DEX-fructose (60% higher than CTL-SC and 73% higher than DEXA-SC; *p* < 0.01) (Figure 5C).

The content of GLUT5 in the jejunum of the 40 h fasted rats was also influenced by in utero exposure to DEX (*p* = 0.0299). The post hoc analysis revealed a specific increase of GLUT5 content in the jejunum of the 40 h fasted DEX-fructose (75% higher than CTL-fructose; *p* < 0.05) (Figure 5D).

### 3.6. Rats Born to DEX-Treated Mothers That Consume Fructose during Adulthood Display Morphological Changes in the Jejunum Epithelium

**Figure 6:**
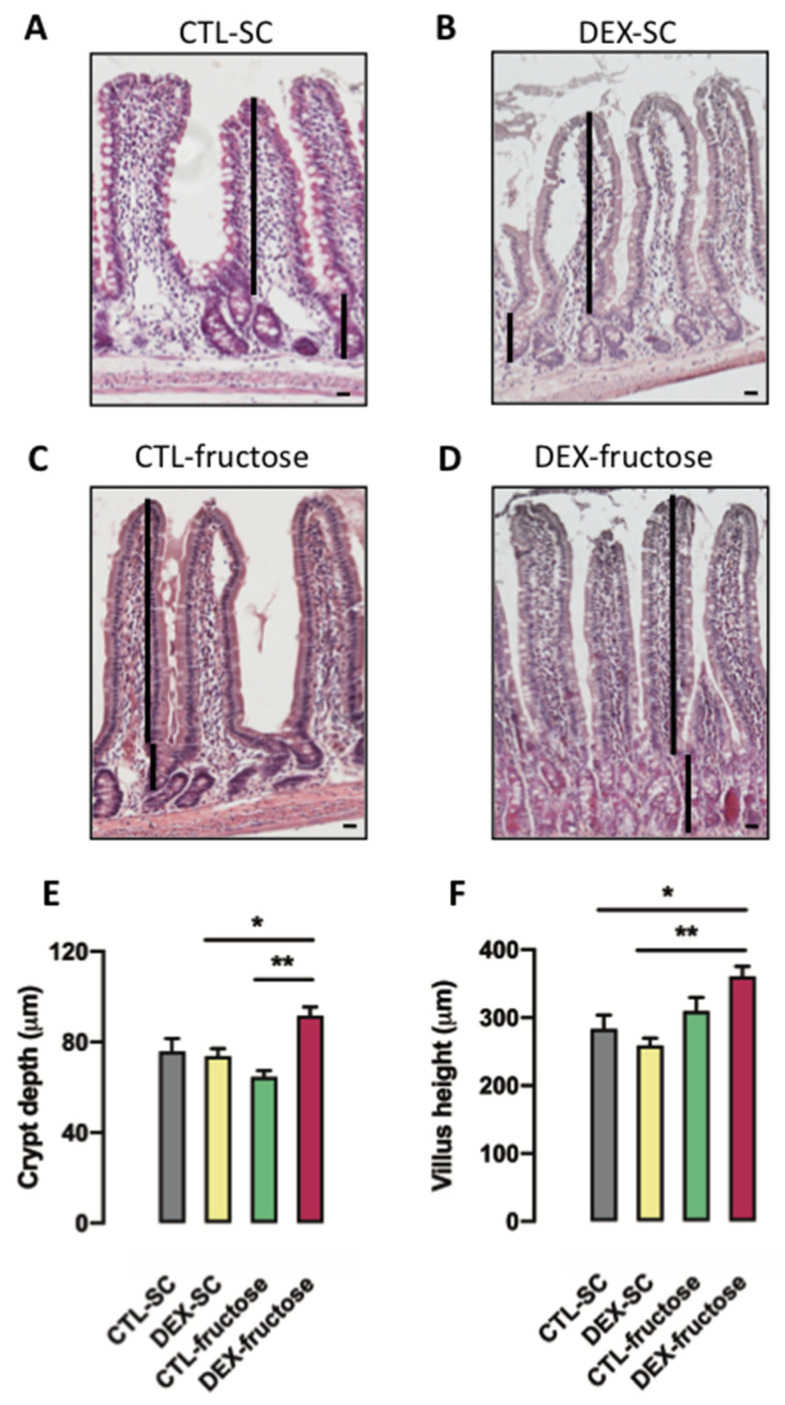
Morphometric analysis of the jejunum wall. The figure shows representative sections of villus and crypts of the four experimental groups (**A**–**D**). The height of each villus was measured from the top of the villus to the crypt transition (**E**), and the crypt depth was defined as the invagination between two villi (**F**). Results are presented as mean ± standard error of the mean (S.E.M.). * *p* < 0.05, ** *p* < 0.01 (*n* = 5). Offspring born to control (CTL) mothers that received only standard chow (SC) and tap water during adult life (CTL-SC); offspring born to dexamethasone (DEX)-treated mothers that received only SC and tap water during adult life (DEX-SC); offspring born to CTL mothers that received SC plus 10% fructose during adult life (CTL-fructose); and offspring born to DEX-treated mothers that received SC plus 10% fructose during adult life (DEX-fructose).

We have also assessed the mean crypt depth and the mean villous height, two aspects of the jejunum epithelium that are vital for its absorptive capacity. Images of the HE-stained jejunum sections are shown from each of the four different groups of 40 h fasted adult offspring (Figure 6A–D).

The mean crypt depth in the jejunum epithelium of 40 h fasted rats was modulated by in utero exposure to DEX (*p* = 0.0119). On the other hand, the post hoc analysis revealed that crypt depth was only increased in the jejunum epithelium of 40 h fasted DEX-fructose rats (25% higher than DEX-SC and 43% higher than CTL-fructose; *p* < 0.05 and *p* < 0.01) (Figure 6E).

Villous height was influenced by treatment with 10% fructose during adulthood (*p* = 0.0017). Similar to crypt depth, the post hoc analysis revealed that villous height was only increased in the jejunum epithelium of 40 h fasted DEX-fructose (27% higher than CTL-SC and 39% higher than DEX-SC; *p* < 0.05 and *p* < 0.01) (Figure 6F).

**Figure 7:**
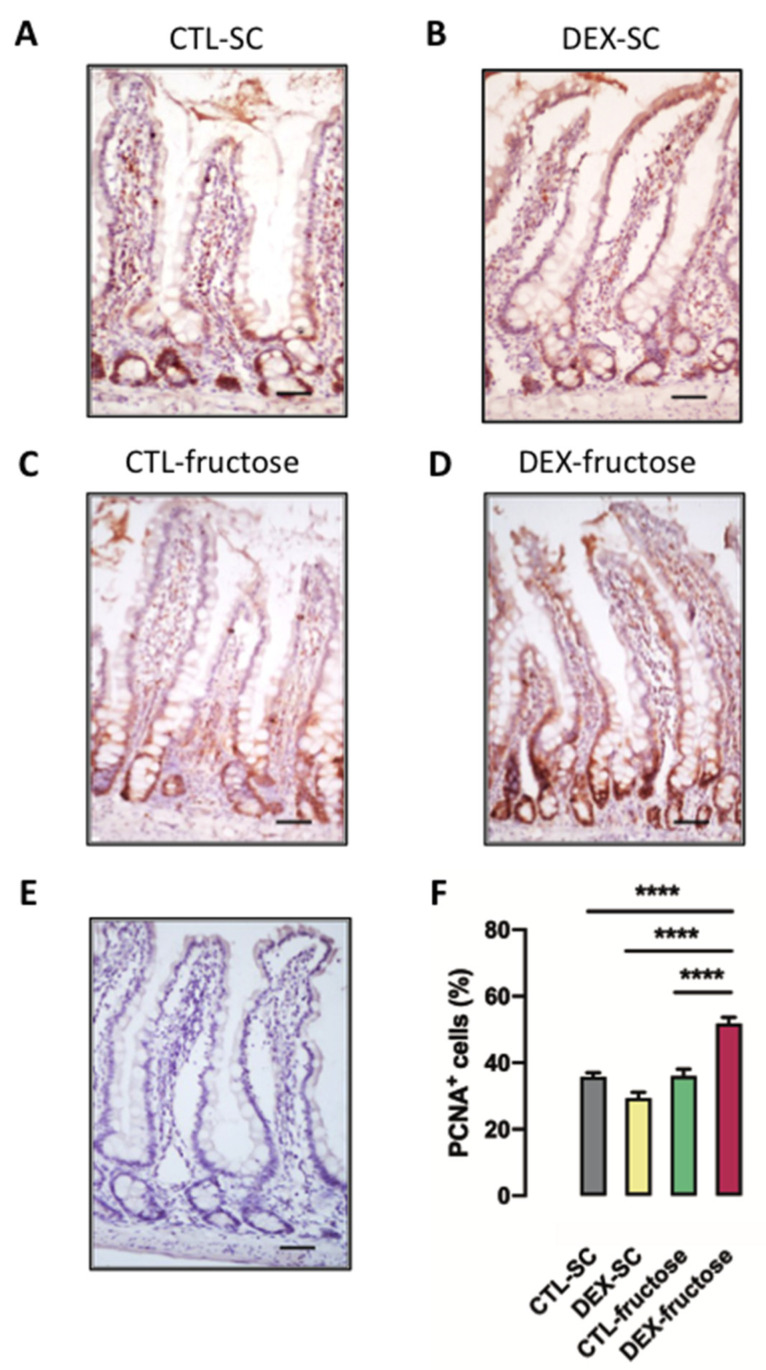
Jejunum was removed for immunohistochemical detection of proliferating cell nuclear antigen (PCNA). The figure shows representative sections of PCNA staining (**A**–**D**) and negative control sample (**E**). Sections were used to calculate the percentage of PCNA-positive cells in crypt cells (**F**). Results are presented as mean ± standard error of the mean (S.E.M.). **** *p* < 0.0001 (*n* = 5). Offspring born to control (CTL) mothers that received only standard chow (SC) and tap water during adult life (CTL-SC); offspring born to dexamethasone (DEX)-treated mothers that received only SC and tap water during adult life (DEX-SC); offspring born to CTL mothers that received SC plus 10% fructose during adult life (CTL-fructose); and offspring born to DEX-treated mothers that received SC plus 10% fructose during adult life (DEX-fructose).

The proliferative potential in the jejunum epithelium was evaluated by assessing the relative number of PCNA-positive cells. Representative images of the jejunum sections stained with anti-PCNA antibody are shown from each of the four different groups of 40 h fasted adult offspring (Figure 7A–D). The hematoxylin-counterstained section that served as a negative control (by omission of the primary antibody) is shown in Figure 7E.

Both factors, in utero exposure to DEX and treatment with 10% fructose during adulthood, influenced cell proliferation rate in the jejunum epithelium of the 40 h fasted rats (*p* = 0.0158 and *p* < 0.0001, respectively). The post hoc analysis revealed a particular increase in the cell proliferation rate of the jejunum epithelium of 40 h fasted DEX-fructose (48% higher than CTL-SC, 78% higher than DEX-SC, and 43% higher than CTL-fructose; *p* < 0.0001) (Figure 7F).

## 4. Discussion

In utero exposure to DEX is well known for programming metabolic changes in the adult offspring of rats. The metabolic imprinting caused by excessive exposure to DEX during fetal life is hallmarked by glucose intolerance, increased whole-body gluconeogenesis, and upregulation of PEPCK expression in the liver [17,33,34]. Recently, we have also described that in utero exposure to DEX exacerbates hepatic steatosis caused by fructose consumption during adult life [16]. The present study further contributes to this topic by revealing that rats born to DEX-treated mothers present exacerbated intestinal gluconeogenesis after consuming excessive fructose during adult life.

Changes in key endpoints support the above claim; increased PEPCK expression and activity in the jejunum and increased portal glucose levels were detected after a 40 h fasting in rats born to DEX-treated mothers that consumed fructose during adult life. An additional finding that supports the proposition that in utero exposure to DEX increases gluconeogenesis capacity is the higher portal glucose levels detected in DEX-fructose rats 90 min after the challenge with pyruvate, a known gluconeogenesis substrate. The 40 h fasting that preceded our sample collection and the PTT was performed because it has been previously described that intestinal gluconeogenesis does not significantly occur during shorter periods of food deprivation [25].

Another finding that supports the notion that intestinal gluconeogenesis is increased in rats born to DEX-treated mothers that consume fructose during adulthood is the increase in GLUT2 content in the jejunum of DEX-fructose rats, with parallel reduction in HK activity. GLUT2 is classically recognized for mediating the basolateral transport of glucose to the capillary vessels that feed the portal vein [35]. Our interpretation is that enterocytes of DEX-fructose rats have increased ability to synthesize glucose de novo (due to increased PEPCK) and release the newly synthesized glucose to the portal bloodstream (due to increased GLUT2 expression). Lower HK activity in the enterocytes of the DEX-fructose rats may contribute to the increased intestinal glucose release by reducing their rate of conversion of newly synthesized glucose back to glucose-6-phosphate.

In parallel with the above-mentioned biochemical changes that evidence increased intestinal gluconeogenesis, DEX-fructose rats exhibited lower portal lactate levels. This is particularly relevant because 10% fructose solution was replaced by water during the 40 h fasting. Thus, the current experiments do not support the notion that in utero exposure to DEX increases intestinal conversion of fructose into glucose but instead indicate that the jejunum of 40 h fasted adult DEX-fructose rats may increase the use of lactate as substrate for gluconeogenesis.

Interestingly, previous studies have reported that the small intestine of the fasted adult rat preferentially uses glutamine and glycerol, instead of lactate, as substrates for gluconeogenesis [23]. On the other hand, the small intestine of suckling rats is able to convert lactate into glucose [36]. Considering this, our data suggest that the small intestine of the offspring born to DEX-treated mothers that chronically consume fructose during adult life preserves a metabolic feature of the newborn small intestine.

Although it is challenging to presume the functional relevance of the increased GLUT5 in the jejunum of DEX-fructose rats after a 40 h fasting, this particular result reinforces the proposition that the small intestine of the adult offspring born to DEX-treated mothers preserves phenotypic features of the newborn after chronic exposure to fructose. Supporting this suggestion, it was previously demonstrated that DEX exacerbates GLUT5 expression induced by fructose in samples of small intestine of neonatal rats [37]. It is important to note that in utero exposure to DEX alone is not sufficient to stimulate GLUT5 content in the jejunum of the offspring. Such findings have also been previously reported in other studies [38] and support the notion that the two factors together (both in utero exposure to DEX and fructose consumption) seem to be necessary for enhancing jejunal GLUT5 content.

With regard to the morphological impact of DEX on the small intestine early in life, it has been previously reported that the lactating pups treated with DEX during lactation exhibit transitory changes in the small intestine epithelial architecture. Pups treated with DEX between the 11th and the 21st days of life display increased villous height and crypt depth in the jejunum soon after weaning. These changes are no longer detected by the age of 50 days [39]. Hence, we conclude that aside from the metabolic/biochemical features, morphological changes transiently described in the lactating pups exposed to DEX during early life are sustained in the adult offspring born to DEX-treated mothers only after consumption of excessive fructose.

We have also found that the frequency of PCNA-positive cells in the jejunum epithelial surface, a parameter that spontaneously reduces in the jejunum of the adult rat as the age advances beyond 90 days of life [40], is exacerbated in the 160-day-old DEX-fructose rats. Instead, *Ggt1* expression, an enzyme that plays a crucial role in de novo synthesis of intracellular GSH and ROS removal [41], is increased in the jejunum of adult rats born to DEX-treated mothers, irrespective of fructose consumption. Notably, GGT1 inhibition was associated with increased apoptosis in smooth muscle cells [42]. Thus, intestinal epithelial cells of the adult DEX-fructose offspring are unique in such a way that they combine long-term pro-proliferative and antiapoptotic adaptations.

Another interesting metabolic feature exhibited in the 40 h fasted DEX-fructose rats is the increased circulating triglyceride levels. Although we are not able to discern the hepatic or the intestinal origin of the lipoproteins that contribute to this phenomenon, it is important to take into account that intestinal production of triglyceride-enriched lipoprotein accounts for up to 40% of the triglycerides in fasting rats [43]. Moreover, chronic consumption of a fructose-enriched diet was described to increase the intestinal production of chylomicrons during fasting periods [44].

In summary, the present study supports the proposition that consumption of fructose by adult rats exposed to DEX during fetal life leads to an exacerbation in intestinal gluconeogenesis and retention of morphological features in the jejunum that are commonly found during neonatal life. These data provide a new mechanism to explain the increased prevalence of metabolic disturbances in humans that are born with low birth weight.
